# Neuromodulatory Effects of Transcranial Pulse Stimulation (TPS) in Neurological and Psychiatric Disorders—A Systematic Review and Meta-Analysis

**DOI:** 10.3390/neurolint17110188

**Published:** 2025-11-18

**Authors:** Selma Polte, Larissa Klingmann, Anna Seßmann, Svenja Schwichtenberg, Christoph S. Herrmann, Karsten Witt, Mandy Roheger

**Affiliations:** 1 Ambulatory Assessment in Psychology, Department of Psychology, Carl von Ossietzky Universität Oldenburg, 26129 Oldenburg, Germany; larissa.klingmann@uni-oldenburg.de (L.K.); anna.sessmann@uni-oldenburg.de (A.S.); mandy.roheger@uni-oldenburg.de (M.R.); 2 Department of Neurology, Carl von Ossietzky Universität Oldenburg, 26129 Oldenburg, Germany; svenja.schwichtenberg@uni-oldenburg.de (S.S.); karsten.witt@uni-oldenburg.de (K.W.); 3 University Clinic for Neurology at the Evangelical Hospital, 26129 Oldenburg, Germany; 4 Experimental Psychology Lab, Department of Psychology, Carl von Ossietzky Universität Oldenburg, 26129 Oldenburg, Germany; christoph.herrmann@uni-oldenburg.de; 5 Research Center Neurosensory Science, Carl von Ossietzky Universität Oldenburg, 26129 Oldenburg, Germany

**Keywords:** transcranial pulse stimulation, low-energy focused ultrasound stimulation, systematic review, neurological disorders, psychiatric disorders

## Abstract

Background: Transcranial pulse stimulation (TPS) is an innovative non-invasive brain stimulation technique using ultrasonic waves. Despite its application in first clinical trials, so far, no systematic overview of its effects across different patient populations has been conducted. Objectives: This systematic review and meta-analysis examines the effects of TPS on cognitive, motor, and mental health outcomes as well as on patient safety in neurological and psychiatric disorders. Methods: We conducted a literature search in MEDLINE, PsycINFO & PsycArticles, CENTRAL, Web of Science, and Google Scholar, covering the period from January 2013 to December 2024. Two independent reviewers conducted the study selection, data extraction, and quality assessment. To evaluate the risk of bias, the RoB2 tool was used for randomized studies and the ROBINS-I tool for non-randomized studies. Results: A total of fifteen studies (five randomized controlled trials and ten non-blinded, single-arm trials) including both adolescent and adult and elderly patient populations (Alzheimer’s disease, mild cognitive impairment, Parkinson’s disease, major depressive disorder, autism spectrum disorder, attention-deficit hyperactivity disorder) were included. Positive effects of TPS intervention on cognitive, motor, and mental health outcomes, as well as a high safety profile, were demonstrated in a majority of the studies and outcome parameters. However, limitations of the included studies persist due to small sample sizes, lack of control groups, retrospective analyses, and heterogeneity of study protocols and measurements. Conclusions: TPS is a safe and promising method for treating neurological and psychiatric disorders. To better assess the potential of this innovative technique, standardized protocol procedures and larger, sham-controlled trials are needed.

## 1. Introduction

Non-invasive neuromodulatory technologies are a rapidly growing field of research [1]. Due to their ability to modulate synaptic plasticity [2], they achieve effects that outlast the end of stimulation and are becoming increasingly important for clinical therapy [3,4]. However, transcranial magnetic stimulation (TMS) and transcranial direct current stimulation (tDCS) can only achieve limited depth effects when inducing magnetic or electrical currents, and tDCS can only achieve relatively low spatial resolution [5,6]. In contrast, transcranial ultrasound stimulation (TUS) travels through brain tissue as waves, thereby achieving high spatial resolution and greater depth penetration. Thus, even deep brain structures such as the amygdala, the thalamus, or the anterior cingulate cortex can be reached using TUS [7]. The spatial resolution in the order of 1–10 mm enables precise applications and can be integrated with advanced focusation and neuronavigation systems, including real-time feedback of stimulation effects on individual brain images, which positions TUS for broad clinical application [8]. For example, recent TUS research has focused on targeting specific subdivisions within the nuclei, such as the striatum or regions of the basal ganglia, which are particularly relevant in the field of psychiatric conditions [9]. Just like diagnostic ultrasound, which has been used ubiquitously in everyday clinical practice for many decades, therapeutic ultrasound has proven to be a safe technology with only mild to moderate side effects (i.e., headache, mood deterioration, scalp heating, cognitive problems, neck pain, muscle twitches, anxiety, sleepiness, and itchiness) [10,11,12].

Generally, sound waves above the audible range are referred to as ultrasonic waves. They have a frequency between 100 kHz and 100 MHz. There are different subtypes of transcranial ultrasound, which will be shortly introduced in the following paragraphs. The diagnostic ultrasound used in everyday clinical practice requires high US frequencies in the 2–15 MHz range for clear image display. Higher sound frequencies yield better image resolution and allow for a smaller, more precise focal point. In contrast, lower frequencies result in reduced resolution and a larger, less precise, focal point. Utilizing focused ultrasound (FUS), different therapeutic effects can be achieved depending on the applied intensities [13]. Using high-intensity focused US (HIFU), localized tissue heating can be attained, which leads to the denaturation of proteins and thus a permanent focal tissue defect (ablation) [14]. This is, for example, being used in the FDA-approved thalamotomy for essential tremor [15]. Low-intensity focused US (LIFU) has more complex effects on tissue, including opening the blood–brain barrier [16] or inducing neuromodulatory excitatory or inhibitory effects. These effects may involve mechanical deformation of cell membranes, altered membrane permeability, activation of mechanosensitive channels, modulation of synaptic vesicles, sonoporation (use of ultrasound to transiently heighten permeability of the cell plasma membrane), slight temperature increases, and intra-neuronal microtubule resonance [17].

Subtypes of LIFU are ultrasound stimulation with theta burst (tbTUS) and transcranial pulse stimulation (TPS). The tbTUS protocol uses a burst frequency of 5 Hz, which is modeled on the frequency of the theta rhythm of the hippocampus and is characterized by long burst durations (tone burst duration = 20 ms) [18]. In contrast, TPS uses singular ultrashort (3 µs, corresponding to 3333 kHz) shock waves with a repetition frequency of 1–5 Hz (no sine wave) and an intensity of 0.1 W/cm^2^ [19]. Table 1 provides an overview of the above-described subtypes of TUS.

TPS is the first non-invasive brain stimulation (NIBS) technique using ultrasound that has received the CE mark for clinical applications [20]. In contrast to other FUS techniques, its ‘waves’ consist of a large but brief positive peak, i.e., 3 µs, followed by a smaller but longer-lasting negative amplitude. This stimulation protocol achieves charge balancing and is thought to reduce the risk of side effects, such as tissue heating and opening of the blood–brain barrier [21]; standing waves; and secondary stimulation maxima. Furthermore, better penetration through the skull could be achieved due to low US frequencies [19,22]. Although the exact biological mechanisms remain unknown, the therapeutic effects of TPS appear to originate from the interaction of ultrasound pulses with the brain tissue. It is assumed that ultrasound shock waves interact with the cell membranes of nerve and glial cells, thereby influencing their electrical conductivity properties via mechanical deflection [19]. In addition, the activity of mechanosensitive membrane channels and receptors is modulated. Ultrasound application also affects gene expression, increasing extracellular dopamine and serotonin concentrations while reducing extracellular GABA concentrations [20]. Overall, this increases the excitability of nerve tissue, as evidenced by a dose-dependent amplification of the cortical sSEP signal [20]. Diffusion tensor imaging has demonstrated lower axial diffusivity in the white matter of healthy subjects following TPS stimulation [20], reflecting either an increased density of stimulated brain fibres or enlarged axonal calibers. At the network level, connectivity improves between functionally relevant areas in both healthy subjects [20] and Alzheimer’s patients [20].

**Figure 1:**
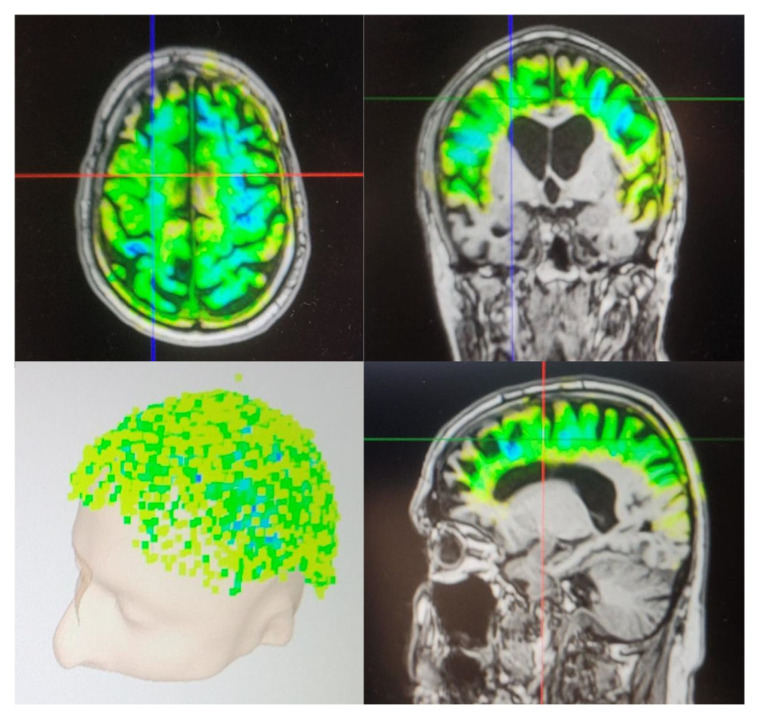
Visualization of TPS after one session.

Figure 1 is an example of the visualization of TPS after one session. Individual neuro-navigation is calculated before each session for visualization purposes. The color indicates the amount of energy calculated to be applied to the respective area. The darker the color, the more energy was delivered. The location of the delivered impulses is shown in the left lower quadrant.

Studies on patients with Alzheimer’s disease (AD) have suggested the potential of TPS as an effective and safe add-on therapy, which may be administered concurrently with established treatment methods, making it particularly promising in a therapeutic context [19,23,24]. So far, the feasibility of TPS has been shown in the first clinical studies focusing on patients with AD [19,22,24], Parkinson’s disease (PD) [25], major depressive disorder (MDD) [26], autism spectrum disorder (ASD) [27], and attention-deficit hyperactivity disorder (ADHD) [28]. Yet, a systematic overview and detailed, summarized description of TPS studies in clinical populations is missing so far.

Thus, this systematic review will be the first to give a systematic overview of the neuromodulatory effects of TPS in neurological and psychiatric disorders. Effects will be categorized in neuroimaging, neurophysiological, and clinical–behavioral outcomes. Clinical–behavioral outcomes will be further differentiated into cognitive, motor, and mental health outcomes. Additionally, patient outcome measures in the form of adverse events will be assessed.

Given the early stage of adoption and clinical use of TPS, available studies often include heterogeneous patient populations. In this review, we deliberately summarize findings across multiple conditions to provide a comprehensive review, while recognizing that disorder-specific reviews will be needed in the future.

## 2. Materials and Methods

For this review, we followed the Preferred Reporting Items for Systematic Reviews and Meta-Analyses (PRISMA) [29] method. It was pre-registered on 26 November 2024 on Prospero (CRD42024619018). The methodology is described in more detail in the following section. The completed PRISMA checklist [30] is provided in the Supplementary Materials (Table S1).

### 2.1. Eligibility Criteria

We included both controlled and uncontrolled experimental studies in our review. Eligible publications were selected based on the availability of sufficient details regarding study design, participant characteristics, and interventions, as outlined in the full text or abstract. Trials were included if they involved participants of all ages with a confirmed diagnosis of a neurological or psychiatric disorder. We considered all trials employing TPS interventions for neuromodulation, irrespective of the stimulation target, session frequency, or intervention duration. Based on a prior review on the effectiveness and safety of TUS neuromodulation [12], primary outcome parameters were neurophysiological outcomes, neuroimaging outcomes, and clinical–behavioral outcomes. Clinical–behavioral outcomes are defined as performance in clinical tests, tasks, and scores, and were categorized into cognition, motor function, and mental health. These outcome parameters are especially important to study due to their influence on the daily living of patients with various neurological and psychiatric disorders, as shown in previous studies [31,32,33,34]. Cognitive functions were further classified into the five cognitive domains: visuo-spatial abilities, memory, executive functions, language, and attention. Motor function outcomes are differentiated in severity of motor function, freezing of gait, and functional mobility and balance, based on Ernst et al. [35]. Mental health outcomes were assessed by changes in disease-specific symptoms. As an additional outcome, patient safety evaluations (adverse effects) are included, taking into account that TPS is still a novel brain stimulation technique [19].

### 2.2. Search Methods for Identification of Studies

#### Electronic Searches

We conducted a systematic search for articles in English and German on 2 December 2024. An updated search was conducted on 19 May 2025. Only studies from 1 January 2013 onwards were included, since there were no TUS studies conducted on humans before that. For a comprehensive review, databases focused on biomedical research (MEDLINE), psychological research (PsycINFO & PsycArticles), and the Cochrane Central Register for Controlled Trials (CENTRAL), as well as two generic databases (Web of Science Core Collection, Google Scholar). The chosen databases were searched with the following search string: ((“transcranial pulse stimulation” OR “TPS” OR “transcranial ultrasound pulse stimulation” OR “ultrasound neuromodulation” OR “transcranial stimulation”) AND (“Alzheimer’s disease” OR “Parkinson’s disease” OR “depression” OR “autism” OR “ADHD” OR “neurocognitive disorders” OR “cognitive impairment” OR “neurodegenerative diseases” OR “mild cognitive impairment” OR “attention deficit hyperactivity disorder” OR “neuropsychiatric disorders”) AND (“clinical trial” OR “randomised controlled trial” OR “RCT” OR “pilot study” OR “observational study” OR “neurology” OR “neurophysiology” OR “neuropsychology”)) NOT (systematic review OR meta-analysis). Search strings for all of the corresponding databased are listed in the Appendix A.

Reference lists of relevant articles were searched to identify further literature. If full texts were not available, the article’s authors were asked to provide the full text within two weeks.

### 2.3. Data Collection and Analysis

#### 2.3.1. Data Extraction (Selection and Coding)

Search results from the predefined databases were downloaded, and duplicates were removed. Afterwards, two reviewers [SP; either LK or AS] independently assessed the titles and abstracts to determine whether they met the inclusion and exclusion criteria. In case of disagreement, the full texts were reviewed. If consensus could not be reached after full-text screening, a third reviewer [either LK or AS] was consulted to resolve the disagreement. This data selection process was conducted in line with the PRISMA statement [29]. Data extraction was conducted by two reviewers using a standardized extraction form based on the Cochrane Data Extraction Form [36], with the information recorded in an Excel spreadsheet. If required, the authors of specific studies were contacted for additional information.

##### Assessment of Risk of Bias in Included Studies

For randomized controlled trials, the updated Cochrane tool (version 2019) for evaluating the risk of bias [36] was used by two independent reviewers [SP; either LK or AS]. If they did not reach an agreement, a third reviewer [either LK or AS] was involved to resolve the issue. The RoB 2 tool assesses bias arising from the randomization process, bias due to deviations from intended interventions, bias due to missing outcome data, bias in measurement of the outcome, and bias in selection of the reported results. The potential risk-of-bias assessments are low risk of bias, some concerns, and high risk of bias.

For non-randomized studies, the ROBINS-I tool (version 2024) [36] was used by two independent reviewers [SP; either LK or AS]. If they were unable to reach an agreement, a third reviewer [again either LK or AS] was consulted. The ROBINS-I tool evaluates bias due to confounding, bias in the selection of participants, bias in the classification of interventions, bias due to deviations from intended interventions, bias due to missing data, bias in measurement outcomes, and bias in reported data. The potential risk-of-bias assessments are low risk of bias, moderate risk of bias, and serious risk of bias.

For adverse effects, it was not possible to perform a formal risk-of-bias assessment. As a result, we made an information evaluation of the risk of bias for this outcome.

## 3. Results

### 3.1. Search Results

**Figure 2:**
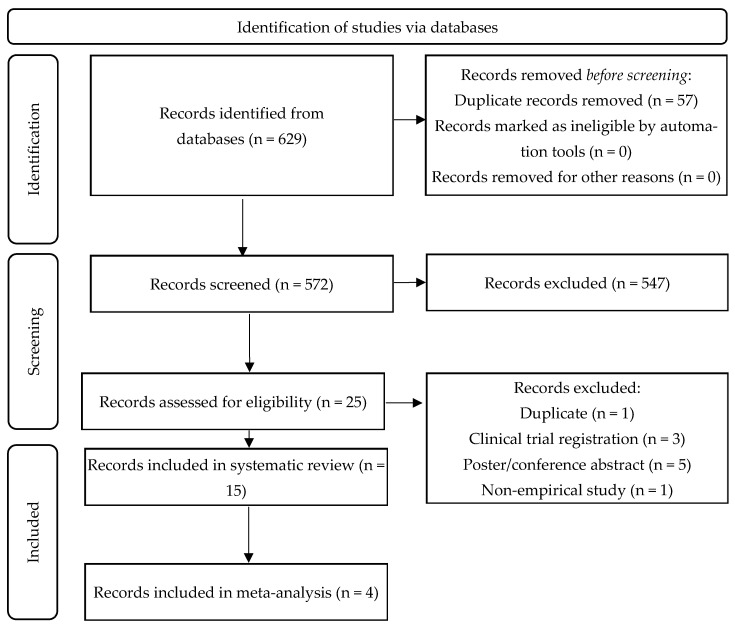
PRISMA diagram of the study selection process.

The search strategy led to a total of 626 articles. After removing duplicates, 569 articles were screened for eligibility based on their titles and abstracts. Of these, 547 were excluded. A further review of 25 full-text articles resulted in the inclusion of 15 articles in the systematic review and the inclusion of four articles in the meta-analysis (see Figure 2). The PRISMA flow diagram in Figure 2 outlines the study selection process.

### 3.2. Characteristics of Included Studies

**Figure 3:**
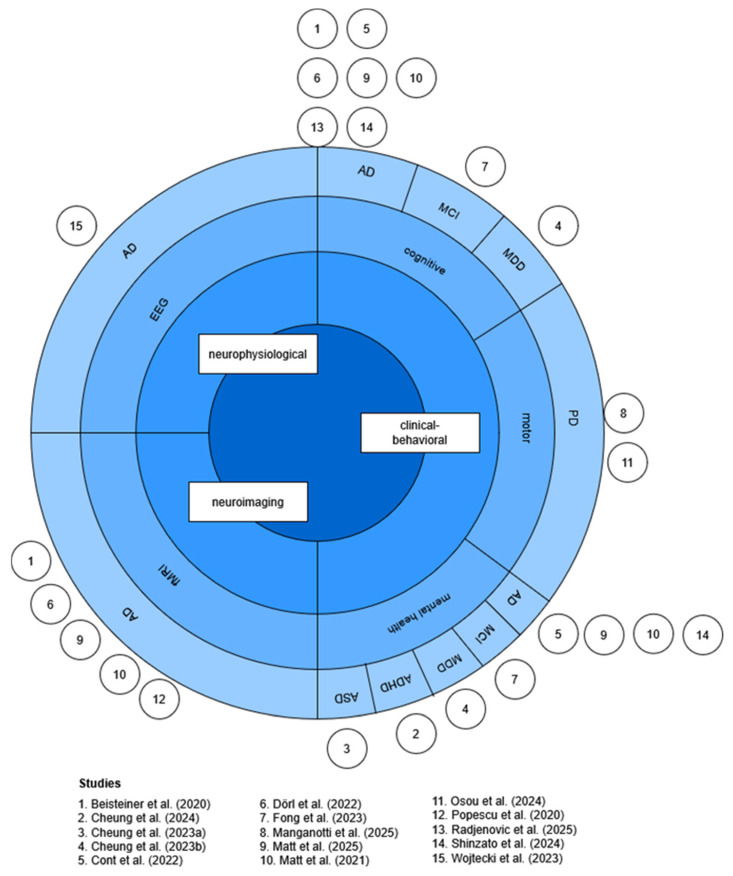
Sunburst chart of outcome measures and patient disorders. Inspired by Sarica et al. [12]. Figure created by the author. Abbreviations: AD = Alzheimer’s disease, ADHD = attention-deficit hyperactivity disorder, ASD = autism spectrum disorder, EEG = electroencephalogram, fMRI = functional magnetic resonance imaging, MDD = major depressive disorder, MCI = mild cognitive impairment, PD = Parkinson’s disease [11,19,20,23,24,25,26,27,28,37,38,39,40,41,42].

Fifteen studies investigating the neuromodulatory effects of TPS were included in this review. Four of the studies [19,20,24,36] were based on the same dataset, which was first published in Beisteiner et al. [19]. Studies were categorized into outcome measures (neuroimaging, neurophysiological, and clinical–behavioral) and patient populations, as visualized in Figure 3.

A detailed overview of included studies with information on patients (i.e., sample sizes, age, sex, diagnosis), TPS procedure (protocol, duration, stimulation target), and side effects is presented in Table 2. The indicated brain regions were stimulated sequentially in every session of the protocol in these studies, which included several studies.

### 3.3. Ongoing Clinical Trials

Considering that TPS is still a novel technique, there are currently six registered clinical trials targeting TPS intervention in neurological or psychiatric disorders (https://clinicaltrials.gov/ accessed on 10 November 2025) that are currently recruiting. Trial numbers, patient diagnoses, and primary outcome measures are listed in Table 3.

### 3.4. Systematic Review

#### 3.4.1. Alzheimer’s Disease

Currently, CE certification covers TPS for the treatment of patients with AD in Europe only. Consequently, most of the studies have focused on the application in this patient group. Brain stimulation followed a global approach targeting AD-relevant networks, including the frontal lobe, parietal lobe, temporal lobe [11,19,20,23,24,36,37,42], and occipital lobe [40]. One study [11] also targeted the hippocampus and, therefore, a deep brain region.

Eight of the studies on patients with AD [11,19,20,23,24,36,37,42] conducted an intervention with six to twelve TPS sessions (two to three sessions per week) over a period of two to five weeks, while one study [41] investigated the effects of a single TPS session.

##### Clinical–Behavioral Outcomes

Eight studies [11,19,20,23,24,36,37,42] have investigated clinical–behavioral outcomes in patients with AD. Both cognitive outcomes and mental health outcomes were assessed.

Cognitive Outcomes

Seven studies (five non-blinded, single-arm trials, one retrospective analysis, one randomized controlled trial) [11,19,20,23,24,36,37,42] with a total of 209 patients with AD focused on the cognitive outcomes of TPS intervention. As pointed out before, the studies of Dörl et al. [37] and Popescu et al. [24] are based on the same dataset first published in Beisteiner et al. [19]; therefore, only the latter one will be considered in the comparison of cognitive outcomes presented in Table 4.

Beisteiner et al. [19], Radjenovic et al. [11], and Cont et al. [23] found a significant improvement in global cognition from baseline to poststimulation, measured with the Consortium to Establish a Registry for Alzheimer’s Disease (CERAD) total score and the Alzheimer’s Disease Assessment Scale (ADAS) subscale for cognition, respectively. These neuropsychological tests were developed to assess cognitive deficits in AD, including memory, language, attention, and other cognitive functions, and are especially suitable to evaluate the severity of AD symptoms. The positive effects found by Beisteiner et al. [19] were also present one month and three months after stimulation, suggesting long-term effects of TPS intervention. However, Cont et al. [23] found no improvements in global cognition measured with the Mini-Mental State Examination (MMSE) and the Montreal Cognitive Assessment (MoCA) in the same study. This variation between the different neuropsychological tests can likely be explained by the different sensitivities of the evaluations [23]. As the MoCA and MMSE are primarily screening instruments, they may not capture the specific cognitive domains assessed by the CERAD with comparable precision and are, therefore, less sensitive to potential changes in TPS.

In contrast, Shinzato et al. [40] and Matt et al. [38], who also applied the CERAD and ADAS subscale for cognition, respectively, did not find significant improvements after stimulation. However, the study by Shinzato et al. [40] differs from the previously mentioned studies in two aspects, which might partly explain this difference. Firstly, Shinzato et al. [40] did not evaluate cognition immediately at poststimulation but only after one and three months after the intervention. Therefore, potential improvements at poststimulation are not accounted for. Secondly, while the previously mentioned studies administered three TPS sessions per week for two to four weeks, Shinzato et al. [40] applied only two sessions per week for five weeks, resulting in a lower frequency/intensity of TPS treatment per week. This might influence the neuromodulatory effects and corresponding cognitive outcomes of the intervention.

Interestingly, the only sham-controlled trial conducted in patients with AD by Matt et al. [38] did not find a significant improvement in overall cognition across the whole sample. However, they found a significant increase in the CERAD score only in the younger subgroup of patients (≤70 years), suggesting that the effect of TPS in cognitively impaired patients might be age sensitive.

Next to global cognition, Beisteiner et al. [19] additionally assessed the specific domains of memory, language, and visuo-spatial abilities through subscales of the CERAD. There were improvements and small effect sizes in the domains of memory and language, but no improvements in visuo-spatial abilities [19]. The absence of a significant effect in visuo-spatial abilities found by Beisteiner et al. [19] is not surprising, as in this study, the occipital-parietal cortex, which is essential for visuo-spatial processing, was not stimulated. Thus, this finding supports the observation that treatment effects are only visible for stimulated regions [19].

Mental Health Outcomes

In addition to cognitive outcomes, two non-blinded, single-arm studies [19,23] and one randomized controlled trial [38], including a total of 94 patients with AD, have investigated the effects of TPS intervention on mental health, specifically on depressive symptoms. Two studies administered the Beck’s Depression Inventory (BDI) [19,37] and one study the ADAS subscale for depressive symptoms [23]. All three studies reported significant reductions. This finding suggests that TPS may not only have a positive effect on cognitive functions but also on mental health, and especially on depressive symptoms in patients with AD (Table 5).

##### Neuroimaging Outcomes

Four studies [19,20,24,36] based on one dataset of 18 patients with AD from a non-blinded single-arm study, as well as one randomized controlled trial [38], investigated the neuroimaging results of TPS intervention. Both Beisteiner et al. [19] and Matt et al. [38] reported increased activation in the precuneus, suggesting functional upregulation of the memory network. Furthermore, Beisteiner et al. [19] found increasing resting-state functional connectivity in the hippocampus, parahippocampal cortex, and parietal cortex, with these changes also significantly correlating with the CERAD cognitive performance scores. In addition, Matt et al. [38] also found increased activation in regions associated with executive and visual processing, as well as an increase in global efficiency within the attention network, suggesting cognitive effects of TPS beyond memory enhancement. Dörl et al. [37] investigated the effects of TPS on the visuo-constructive network, a brain region not stimulated during the intervention. They found a decreased connectivity of the visuo-constructive areas and declined visuo-constructive processing according to neuropsychological test scores, which were in line with the natural progress of the disease. This finding emphasizes the functional specificity of TPS according to the chosen regions for stimulation. Matt et al. [20] found that TPS treatment decreased the functional connectivity between the left frontal orbital cortex and the right anterior insula, and that functional connectivity was positively correlated with BDI-II scores. In other words, higher functional connectivity in these areas, corresponding to a higher interruption of normal connectivity, was associated with more severe depressive symptoms. Popescu et al. [24] investigated the effects of TPS on cortical atrophy in patients with AD. They reported no significant differences in total brain volume between the pre- and post-treatment groups. However, a significant increase in cortical thickness was observed in the left precuneus and the left superior parietal lobe after stimulation, as well as a positive correlation between cortical thickness changes in these brain regions and cognitive changes in the CERAD. Thus, this study shows that TPS might not only have functional effects as previously suggested but might also reduce the atrophy of brain tissue stimulated with ultrasound.

##### Neurophysiological Outcomes

One study [41] investigated the neurophysiological effects of one session of TPS in a sample of ten patients with AD in a non-blinded single-arm study. The brain regions stimulated were the frontal cortex, parietal cortex, temporal cortex, and precuneus. The effects of TPS were investigated through resting-state EEG measurements. The findings suggest significant effects of TPS on changes in power in the frontal and occipital region; in coherence in the frontal, occipital, and temporal regions; in Tsallis entropy in the temporal and frontal regions; and in cross-frequency coupling in the parietal-frontal, parietal-temporal, and frontal-temporal regions [41]. These changes may reflect a direct biological impact of the stimulation of the brain. However, considering that the intervention was applied at 4 Hz, the changes in alpha-to-gamma oscillations might not necessarily indicate a direct activation of neuronal clusters [41]. They could potentially also be related to activation of mechanosensitive ion channels, enhanced metabolic activity, or the discharge of nitric oxide in the targeted brain regions [41].

#### 3.4.2. Mild Cognitive Impairment

One study [42] investigated the effects of TPS in 19 patients with mild cognitive impairment (MCI) in a non-blinded single-arm study. MCI is characterized by a decrease in cognitive functions beyond normal ageing, which may, but does not necessarily have to, progress to dementia [44]. Fong et al. [42] stimulated the same brain areas as in the studies on patients with AD, namely, the frontal, parietal, temporal, and occipital lobes. Likewise, a similar study protocol was followed, including six sessions over two weeks.

Fong et al. [42] found significant improvements in cognitive functions, assessed by the MoCA, Verbal Fluency Test, and the Stroop test. However, there was no effect on mental health outcomes, evaluated by the Hamilton Depressive Rating Scale (HDR-17) and the Apathy Evaluation Scale, suggesting that only the cognitive but not the mental health effects previously seen in patients with AD might translate to this patient group. However, more trials are needed to confirm these findings and assess whether TPS might be a potential therapy option for patients with MCI.

#### 3.4.3. Parkinson’s Disease

Two studies measured the effects of TPS on motor functions in a total of 36 patients with PD, through a non-blinded single-arm study [25] and a non-randomized controlled trial [43], respectively. PD is characterized by a variety of motor symptoms, including akinesia and bradykinesia, or tremor and rigidity, which rely on elemental motor processes [45]. Consequently, the brain regions stimulated were the motor cortex, including the primary sensorimotor area, supplementary motor area, and cingulate motor cortex. While the primary sensorimotor area is involved in the execution of voluntary movements and sensory feedback, the supplementary motor area plays a role in motor planning, coordination, and initiation of complex movements. The cingulate motor cortex contributes to emotionally driven movement, error correction, and motivation. Furthermore, in the study of [25], some additional brain regions were targeted based on the patients’ individual symptomology, i.e., the left dorsal prefrontal cortex for depression [25].

In the retrospective analysis by Osou et al. [25] of a case series of 20 patients with PD with a disease duration of 3 to 148 months, the stimulation protocol included ten sessions over two weeks, and the Unified Parkinson’s Disease Rating Scale (UPDRS-III) was investigated as the primary outcome before and after intervention. UPDRS-III significantly increased, with 7 out of 20 patients improving by at least five points, which indicates clinical improvement. However, it needs to be considered that this study was not sham-controlled, and placebo effects might have influenced the results. While this is true for a majority of the included studies, it might be more relevant in the current trial, since the literature suggests that placebo effects can induce dopamine release in the dorsal striatum, relating to placebo-triggered changes in symptoms of patients with PD [25].

The study by Manganotti et al. [43] included 16 patients with PD who received a single session of TPS treatment. Out of these, a subsample of nine patients was additionally admitted to the sham condition after a wash-out period of 30 days. Resting tremor was evaluated at baseline, directly after stimulation, and 24 h after the stimulation. The results suggest that while tremor was always present at baseline, it was reduced at both follow-up test points. However, there was only a decrease in the amplitude of resting tremor, but not in its frequency.

#### 3.4.4. Major Depressive Disorder

Cheung et al. [26] measured the effects of TPS on 30 patients with major depressive disorder (MDD) in a single-blinded, randomized controlled trial with a 1:1 ratio and a follow-up of three months. MDD is marked by consistently low mood, diminished interest or enjoyment in previously pleasurable activities, recurring thoughts of death, and a range of physical and cognitive symptoms [46]. The brain region stimulated was the left dorsolateral prefrontal cortex (dlPFC). Previous research suggests hypoactivity in the left dlPFC in patients with MDD, causing negative emotional perceptions [26].

The stimulation protocol included six sessions over two weeks, and both mental health and cognitive outcomes were assessed. The reasoning for this is that cognition is often affected in brain disorders, with MDD being no exception. Thus, next to the primary outcome of depression, the secondary outcomes included anhedonia, instrumental activities of daily living, overall cognition, working memory, executive function, and attention. There was a significant intervention effect in all outcomes, except for one test relating to attention (TMT-B), where no effect was found.

#### 3.4.5. Autism Spectrum Disorder

Cheung et al. [27] investigated the effects of TPS on young adolescents with autism (ASD) in a double-blind, randomized, sham-controlled trial including 30 patients in total with a 1:1 ratio and a follow-up of 3 months. ASD is a neurodevelopmental condition associated with challenges in social interaction and communication, along with distinctive repetitive and restricted behaviors [47]. The brain region stimulated was the left dlPFC. This region was chosen based on previous NIBS studies showing that stimulation of the left dlPFC can decrease inattention and hyperactivity [27].

The treatment protocol implied six sessions over two weeks. The intervention consisted of only 800 pulses instead of the commonly applied 6000 pulses per session conducted in adults, since the participants were aged 12 to 17, and only one brain region was targeted. Both disorder-specific outcomes and cognitive outcomes were assessed. The main outcome was the Childhood Autism Rating Scale (CARS), a behavioral rating scale to detect autism and measure its severity, which has previously been used in other non-invasive brain stimulation studies. It covers 15 items in different domains, i.e., relating to people, emotional response, verbal/non-verbal communication, etc. Secondary outcomes included further scales to measure ASD symptoms and severity (Autism Spectrum Quotient, Social Responsiveness Scale, Australian Scale for Asperger’s Syndrome) as well as measures of attention, verbal fluency, inhibition control, working memory, and clinical global impression. An inclusion of this neuropsychological assessment is relevant, since previous studies suggest that patients with ASD show distinct neuropsychological patterns across multiple domains [48]. There was a significant improvement in the primary outcome assessing the severity of autism symptoms, as well as significant improvements in tests in the domains of inhibition control, attention, and clinical global impression from pre- to post-intervention.

#### 3.4.6. Attention-Deficit Hyperactivity Disorder

Cheung et al. [28] researched the effects of TPS on young adolescents with ADHD in a double-blind, randomized, sham-controlled trial, including 30 patients in total with a 1:1 ratio and a follow-up of 3 months. ADHD involves, i.e., difficulty sustaining attention, distractibility, and hyperactive-impulsive actions [49]. The brain region stimulated was the left dlPFC. This region was chosen based on previous NIBS studies showing that stimulation of the left dlPFC can decrease inattention and hyperactivity [28].

The treatment protocol consisted of six sessions over two weeks. Like in the study on patients with ASD [27], a reduced 800 TPS pulses per session were administered, considering that the patients were aged 12 to 17 years. Both disorder-specific and cognitive outcomes were assessed. Assessed outcomes were the Swanson, Nolan, and Pelham Teacher and Parent Rating Scale (SNAP-IV), which measures inattention, hyperactivity/impulsivity, and oppositional defiance; the CGI; the ADHD Rating Scale (ADHD RS-IV); and measures of executive function and working memory.

The results suggest significant improvements in ADHD-related symptoms measured by the SNAP-IV and the ADHD RS-IV, as well as in executive functions and some measures of working memory and CGI from pre- to post-intervention.

#### 3.4.7. Adverse Events

Nine studies [11,19,23,25,26,27,28,37,38,41] registered adverse events of the TPS intervention. Patients’ self-reports at each visit were acquired by all six of these studies. Additionally, four studies applied checklists to systematically monitor symptoms [26,27,28,38]. Two studies also evaluated the experienced pain and pressure during treatment using a visual analog scale (VAS; 0 = none and 10 = very strong/pressure/pain) [19,25]. Beisteiner et al. [19] also checked for intracerebral pathologies by inspecting anatomical MRIs, T2*, and FLASH images after each MRI session.

There were no severe side effects reported in any study. Three studies reported that no adverse events were experienced, while the other five studies reported a prevalence of 9% to 65% of patients with mild or transient side effects, including headache, mood deterioration, pain in the jaw, feeling of nausea and drowsiness, depression, anxiety, sleep disorder, and fatigue.

### 3.5. Risk of Bias Assessment

**Figure 4:**
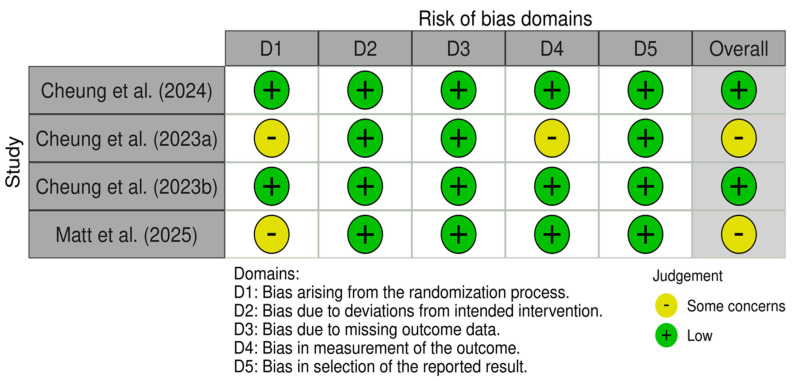
RoB assessment of the randomized studies using the RoB2 tool [26,27,28,40].

**Figure 5:**
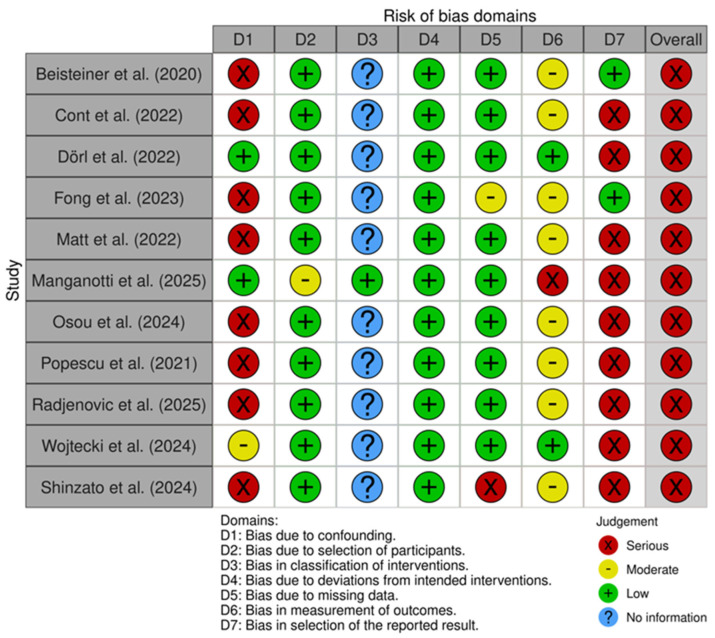
RoB assessment of the non-randomized studies using the ROBINS-I tool [19,20,23,24,25,37,38,39,41,42,43].

The four randomized controlled trials were assessed with the RoB2 tool, while the remaining eleven non-randomized studies were assessed with the ROBINS-I tool. Outcomes of the risk of bias assessment of both tools can be found in Figure 4 and Figure 5, respectively. The overall risk of studies is rated as low risk in n = 2, medium risk in n = 2, and high risk in n = 11 studies. Only one of the nine non-randomized studies included a control group; therefore, domain 3, “Bias in the classification of interventions”, was not assessed in the remaining ten studies. Most of the non-randomized studies showed a high risk in the domain “Bias due to confounding”, as placebo effects were not accounted for. Additionally, many non-randomized studies were rated as medium risk in the domain “Bias in measurement outcomes”, as participants were not blinded, which could have influenced the results, especially when measured through self-reported data, i.e., questionnaires. Finally, many of the non-randomized studies showed a high risk in the domain “Bias in selection of the reported results”, as they did not publish a preregistration or clinical trial registration number.

### 3.6. Meta-Analysis

Originally, it was planned to conduct a meta-analysis for all outcomes; however, due to the limited number of studies, only meta-analyses on the effects of TPS on cognitive functions in patients with AD and on depressive symptoms in patients with AD could be conducted.

#### 3.6.1. Meta-Analysis on TPS Effects on Cognitive Functions in Patients with Alzheimer’s Disease

**Figure 6:**
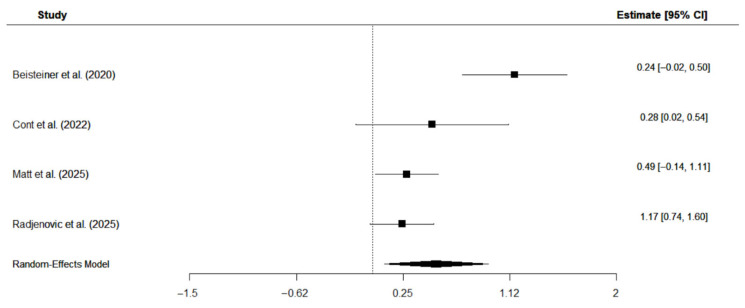
Forest plot on the effects of TPS intervention on overall cognition in patients with AD [19,23,40,43].

Four studies were included in the meta-analysis that investigated the effects of TPS on overall cognition in patients with AD [11,19,23,37]. Three studies [11,19,37] applied the CERAD, while Cont et al. [23] used the cognitive subscale of the ADAS. The analysis employed a random-effects model with the restricted maximum likelihood (REM) estimator to account for the small number of studies and calculated standard mean change, assuming a pre-to-post correlation of r = 0.9, as shown in previous studies on short-term correlations of cognitive tests in patients with AD [50]. Results suggest a moderate positive effect of TPS intervention on overall cognition: Hedge’s g = 0.42, CI [0.15, 0.70], *p* = 0.0024, I^2^ = 59.81%. Individual study effects and the overall pooled effect are displayed in Figure 6.

#### 3.6.2. Meta-Analysis on TPS Effects on Depressive Symptoms in Patients with Alzheimer’s Disease

**Figure 7:**
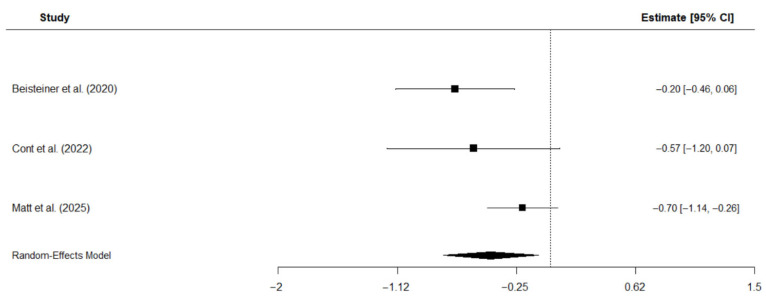
Forest plot of meta-analysis on the effects of TPS on depressive symptoms in patients with AD [19,23,40].

Three studies were included in the meta-analysis that investigated the effects of TPS on depressive symptoms in patients with AD [19,23,37]. Depressive symptoms were assessed with the BDI [19,37] and the subscale for depressive symptoms of the ADAS [23], respectively. The analysis employed a random-effects model with the restricted maximum likelihood (REM) estimator to account for the small number of studies and calculated standardized mean change, assuming a pre-to-post correlation of r = 0.8, as shown in previous studies on short-term correlations of scores assessing depressive symptoms [51]. The results suggest a moderate negative effect of TPS intervention on depressive symptoms: Hedge’s g = −0.44, CI [−0.79, −0.09], *p* = 0.014, I^2^ = 51.6%. This negative effect suggests that TPS reduces depressive symptoms. Individual study effects and the overall pooled effect are displayed in Figure 7.

## 4. Discussion

This review systematically examined the neuromodulatory effects of TPS on neurological and psychiatric disorders reported in the current literature.

A consistent finding across studies is that TPS seems to be a promising intervention, with improvements in cognitive, motor, and mental health outcomes in a majority of the studies and outcome parameters. Positive effects were shown in both adolescent patients aged 12 to 17 (ASD, ADHD), adult patients (MDD), and elderly patients above 50 (AD, MCI, PD). Furthermore, the focality of TPS was demonstrated, with cognitive improvements seen only in domains corresponding to the brain region stimulated. Additionally, six out of twelve studies indicate a long-term effect of TPS intervention, with significant improvements seen up to three months after stimulation. These results highlight the potential of TPS as an add-on therapy in a broad range of clinical populations and across different ages. Across all of the included studies, the stimulation targeted all major cortical lobes, namely the frontal, parietal, temporal, occipital, and limbic regions. The selection of the target regions was based on the results of empirical studies in these areas of the specific disorders. For example, for patients with cognitive impairments (AD, MCI), regions included, i.e., the precuneus cortex and hippocampus, which are often affected by atrophy in these patients [19,39]. Increased activation in these areas (shown by neuroimaging studies) and improvements in related cognitive scores therefore suggest that TPS could potentially help to restore network activity and cognitive functions. Similarly, in patients with PD, TPS was applied to the primary and supplementary motor areas, which typically demonstrate abnormal activity in this patient group, with the aim to support motor function [25]. In the patients with psychiatric disorders (depression, autism, and ADHD), TPS stimulation was targeted at areas related to social cognition and attention (i.e., dlPFC, rTPJ), with the goal of regulating network activity in these regions [26,27,28].

All studies demonstrated a very high safety profile of TPS intervention with no severe side effects but only mild, transient effects. This finding is in line with previous data from animal studies and healthy people [19]. Thus, according to the current evidence, it can be concluded that a TPS protocol of up to 800 pulses per session and a frequency of six sessions over two weeks can be considered safe for adolescent patients and a protocol of up to 6000 pulses per session and a frequency of six to twelve sessions over two to four weeks can be considered safe for elderly patients. However, these findings are primarily about short-term effects and the absence of severe adverse effects. For a better evaluation of TPS safety, future studies should not only consider the mentioned clinical outcomes but also examine the direct and long-term physiological effects of the stimulation. These studies would be helpful to assess whether TPS leads to lasting changes in brain structure or function and to assess safety standards more precisely. For example, it is currently unclear whether a longer or more intense protocol including more than twelve sessions per patient or reoccurring, i.e., monthly, follow-up stimulations would potentially cause any side effects. The initial therapy regimen includes four to six stimulation sessions within the first two weeks of treatment, followed by more variable sessions. Studies exploring different stimulation intensities and frequencies may help to establish a disease-specific standard stimulation protocol. Additionally, it is not investigated yet as to whether TPS could also be a safe and feasible add-on treatment option for children younger than twelve years. Furthermore, the methods used to record adverse effects vary between studies. Having a standardized protocol in place would make it easier to record adverse events systematically and compare different patient populations and stimulation protocols. Finally, the safety of TPS as a potential intervention for patients with other neurodegenerative or psychiatric disorders also needs to be investigated. In particular, the efficacy and safety of combining TPS with new antibody-based therapies should be tested.

Several limitations were found with respect to the included studies. Firstly, four studies relied upon the same dataset, introducing the risk of data redundancy. Secondly, most of the studies included only a small sample size, potentially constraining the reliability of the findings. Thirdly, a majority of the studies did not apply sham stimulation as a control condition, which potentially makes the results susceptible to placebo effects, although this may depend on the clinical population investigated. While Osou et al. [25] pointed out the heightened risk of placebo effects in patients with PD due to dopaminergic mechanisms, Beisteiner et al. [19] suggest that the long-term neuropsychological improvements seen in patients with AD deviate from expected placebo effects. Fourthly, some of the studies conducted retrospective analyses of clinical data that was originally not gathered for research. This caused missing data in some of the tests. Finally, both the measurements for cognitive, motor, and mental health outcomes as well as for adverse events were highly heterogeneous across the studies, making direct comparisons challenging.

To address these limitations, future work should focus on establishing a standardized procedure protocol for TPS. Given the novelty of the technique, there is currently no standardized protocol for TPS or TUS in general. However, the International Transcranial Ultrasonic Stimulation Safety and Standards (ITRUSST) consortium has recently published a practical guide with best practice recommendations for human TUS application [52]. The guidelines address safety and risk management, parameter optimization, equipment and calibration, precise targeting, experimental control, modeling and validation, multimodal integration, and reproducibility. Although addressing the application of TUS in general, they can be used as a framework to inform the development of a standardized protocol for TPS. Particular attention should, thereby, be paid to a standardized brain sonication concept, which refers to a reproducible framework for identifying sonication parameters (i.e., ultrasound operating frequency, pulse duration, pulse repetition frequency, etc.) and target regions across individuals [9]. Developing such a framework could potentially improve reproducibility and comparability of TPS studies, as this review and meta-analysis suggested heterogeneity in TPS procedures and outcomes. However, despite the need for standardization, it should still allow for individual adjustments based on patient status, age group, and individual parameters, such as consideration of skull thickness. This is recommended, since skull attenuation is around 80–90% [38], and variations in skull thickness cause different levels of distortions and scattering of the ultrasonic field between individual persons. Therefore, one approach would be to simulate the transmission of the ultrasonic field through the skull to assess subject-specific effects based on anatomical information derived from neuroimaging [9]. These simulations, based on estimates of skull thickness derived from MRI images, could be further used to improve stimulation protocols. Similar approaches of estimating skull conductivity and compensating for its effects have already been done in EEG and MEG research [53].

Additionally, future research should investigate the effects of TPS in larger, sham-controlled trials. Given that randomized controlled studies are viewed as the ‘golden standard’ in experimental designs, it is surprising that only 5 out of 15 studies have used a sham condition. This is especially unexpected considering that there is an air-filled chamber available within the TPS system that absorbs ultrasound stimulation for sham stimulation. However, the main reason for a missing sham stimulation might be that TPS is already certified for clinical practice in AD, and much of the data has therefore been gathered in the context of therapeutic interventions and only been analyzed for research retrospectively. This might also partly explain the relatively small sample sizes encountered in the non-randomized uncontrolled trials. To address this issue, upcoming trials should be designed in accordance with established research standards, notably including a sham control and an a priori power calculation to determine a sufficient sample size.

Another interesting direction for future research is to investigate the combination of TPS and training, i.e., cognitive training, to see whether it further improves the effects of intervention. Positive effects of combining non-invasive brain stimulation and cognitive training have previously been shown with transcranial direct current stimulation [54] and transcranial alternating current stimulation [55].

Although TPS is already CE-certified for clinical practice in patients with AD, the exact neurophysiological mechanisms of TPS are still not fully understood. There is a need for more basic research focusing on the vascular, metabolic, neurotrophic, electrophysiological, and metaplasticity effects of TPS. Vascular responses refer to the effects on blood vessels and their functions and have been demonstrated in other NIBS, such as transcranial electrical stimulation (tES) [56]. The first evidence points to a link between the effects of TPS on the neurovascular network in an animal model. However, no evidence of tissue heating or changes to the blood–brain barrier has been found [21]. Metabolic effects are physiological changes that alter the uptake and processing of energy-yielding nutrients and have been shown in response to FUS in a previous study [57]. Neurotropic responses are neuronal changes that promote the growth and permanence of nerve cells and have been induced by LIFU, as described before [58]. Neurotropic responses are especially relevant in the field of neurodegenerative diseases, including AD and PD, due to their potential neuroprotective factor [58]. While Wojtecki et al. [41] have provided initial evidence on the electrophysiological effects of TPS using EEG, further research is needed to explain the underlying biological mechanisms of this stimulation technique. Future studies should investigate the electrophysiological correlates of TPS across multiple stimulation sessions and with respect to behavioral outcomes [41]. Finally, meta-plasticity relates to the modulation of synaptic plasticity based on neural activity levels. It has been previously discussed in relation to other NIBS, including TMS, tDCS, and tACS [59] Changes in functional network connectivity [19,57] and cortical grey matter thickness [24] can be interpreted as plasticity-related changes after TPS. Focusing on the potential vascular, metabolic, neurotropic, and meta-plasticity effects of TPS may help to better understand its mechanisms of action and further inform interventions in applied clinical practice. Furthermore, the dose–response relationship of TPS is unclear. One study reported neuromodulatory effects in median nerve somatosensory evoked potentials (SEPs) related to TPS over the primary sensory cortex, demonstrating significant amplitude changes after 10, 100, and 1000 TPS stimuli in a dose-dependent manner [19]. However, more research on the dose–response effects of TPS in a clinical context is needed.

### Strengths and Limitations of This Systematic Review

To the best of our knowledge, this is the first systematic review focusing on the neuromodulatory effects of TPS in neurological and psychiatric disorders. Strengths include the applied methods with a review question based on the PICO system, a systematic literature search across multiple databases, and adherence to the PRISMA guidelines for transparent reporting. Additionally, our review discusses results from different neuroscientific methodologies, including clinical–behavioral outcomes, neuroimaging outcomes, and neurophysiological outcomes.

Limitations must be acknowledged when interpreting the results. One limitation of the present review is the inclusion of a broad range of neurological and psychiatric disorders. This reflects the novelty of TPS and its current limited distribution in clinical studies. While we intentionally summarized all available results across different conditions, future research should focus on disorder-specific investigations. Likewise, due to the novelty of the technique, the systematic review and meta-analysis is based on a very small sample of studies and participants, which may impact the validity of findings with respect to generalizability and statistical power. Due to the limited number of controlled trials (only 5 out of 15), uncontrolled trials were also included in this review, which may adversely affect the quality of the results. Additionally, as there were too few studies assessing the long-term effects of TPS, only short-term effects have been considered in the meta-analyses. Finally, it needs to be noted that the tools used to determine risk of bias are sensitive to subjective evaluations, which may lead to different judgments in other reviews.

## 5. Conclusions

This systematic review suggests that TPS is a promising and safe NIBS technique across a variety of neurological and psychiatric disorders in both adolescent and adult and elderly patients. Positive effects have been found in cognitive, motor, and mental health outcomes in a majority of the studies and outcome parameters. This highlights the potential for clinical practice, especially due to its demonstrated safety. Yet, current studies are affected by small sample sizes, limited sham controls, and a lack of standardized procedures. Future research should prioritize larger, sham-controlled trials; more basic research in the underlying mechanisms of TPS; and the development of a standardized protocol.

## Figures and Tables

**Table 1:** Technologies and terminology in the field of TUS.

Technology/ Terminus	Abbreviation	Frequency	Intensity (I_SPTA_)	Application
Diagnostic US	DUS	approx. 2–15 MHz	<1 W/cm^2^	Diagnostic imaging of anatomical structures
Focused US (FUS)				
High-intensity US	HIFU	approx. 1–7 MHz	100–1000 W/cm^2^	Therapeutic use by creating permanent lesions through thermal destruction of tissue
Low-intensity US	LIFU	200–1000 kHz	<100 W/cm^2^	Therapeutic use by evoking neuromodulatory effects
Transcranial pulse stimulation	TPS	1–5 Hz	0.1 W/cm^2^	
Transcranial ultrasound with theta burst	tbTUS	5 Hz Bursts: 50 Hz	0.23 W/cm^2^	

**Table 2:** Overview of studies included in the systematic review.

Authors	Study Design	Sample Characteristics	TPS Protocol	Duration	Location of Stimulation	Side Effects
Alzheimer’s disease						
Beisteiner et al. (2020) [19] *	NCT	N = 35 (20 females, 15 males); age = 72.38 (7.11); stable antidementia therapy (≥3 months) or none required	EFD = 0.20 mJ/mm^2^, PRF = 5 Hz, 6000 pulses/session	6–12 sessions over 2–4 weeks	dlPFC, IFC, LPC, precuneus cortex, DMN	93% no AEs, 4% headache, 3% mood deterioration
Cont et al. (2022) [23]	NCT	N = 11 (2 females, 9 males); age = 69.82; medication status NR	EFD = 0.20 mJ/mm^2^, PRF = 4 Hz, 6000 pulses/session	6–12 sessions over 2 weeks	PFC, LPC, temporal cortex	73% no AEs, 27 side effects (jaw pain, nausea, drowsiness)
Dörl et al. (2022) [37] *	NCT	N = 18 (11 females, 7 males), age = 69.90; stable antidementia therapy (≥3 months) or none required	EFD = 0.20 mJ/mm^2^, PRF = 5 Hz, 6000 pulses/session	6–12 sessions over 2–4 weeks	dlPFC, IFC, LPC, precuneus cortex, DMN	NR
Matt et al. (2025) [38]	RCT	N = 60 (30 females, 30 males), age = 70.65 (8.16); all on standard antidementia therapy	EFD = 0.2 mJ/mm^2^, PRF = 5 Hz, 6000 pulses/session	6 sessions over 2 weeks	dlPFC, IFC, LPC, precuneus	70% no AEs, 12% depression, 7% headache, 5% dizziness, 2% anxiety, 2% sleep disorder, 2% fatigue
Matt et al. (2022) [20] *	NCT	N = 18 (11 females, 7 males); age = 69.90; stable antidementia therapy (≥3 months) or none required	EFD = 0.20 mJ/mm^2^, PRF = 5 Hz, 6000 pulses/session	6–12 sessions over 2–4 weeks	dlPFC, IFC, LPC, precuneus cortex, DMN	NR
Popescu et al. (2020) [24] *	NCT	N = 17 (gender = NR), age = NR; stable antidementia therapy (≥3 months) or none required	EFD = 0.20 mJ/mm^2^, PRF = 5 Hz, 6000 pulses/session	6–12 sessions over 2–4 weeks	dlPFC, IFC, LPC, precuneus cortex, DMN	NR
Radjenovic et al. (2022) [39]	NCT	N = 58 (26 females, 30 males), age = 71.72 (8.19); all on antidementia medication except one patient only taking dietary supplements	EFD = 0.15–0.25 mJ/mm^2^, PRF = 4 Hz, 2000–4000 pulses/session	10 sessions	dlPFC, IFC, LPC, precuneus, anterior cingulate cortex, hippocampus	81% no AEs, 7% fatigue, 4% transient pain, 3% pressure sensation, <3% dizziness, nausea, confusion, gait disturbances
Shinzato et al. (2024) [40]	NCT	N = 10 (6 females, 4 males); age = NR; on cholinesterase inhibitors and/or memantine at therapeutic doses	EFD = 0.20 mJ/mm^2^, PRF = 5 Hz, 6000 pulses/session	10 sessions over 5 weeks	Frontotemporal	NR
Wojtecki et al. (2025) [41]	NCT	N = 10 (2 females, 8 males), age = 69.20 (7.10); medication status NR	EFD = 0.20 mJ/mm^2^, PRF = 4 Hz, 1004–6000 pulses/session	1 session	PFC, LPC, precuneus cortex, temporal cortex	NR
Mild cognitive impairment						
Fong et al. (2023) [42]	NCT	N = 19 (12 females, 7 males); Age = NR; on stable antidementia therapy ≥ 3 months	6000 pulses/session	6 sessions over 2 weeks	Frontal, parietal, temporal, and occipital lobes	No AEs
Parkinson’s disease						
Manganotti et al. (2025) [43]	NRT	N = 16 (5 females, 11 males), age = 72.63 (8.00); on stable levodopa medication	EFD = 0.2 mJ/mm^2^, PRF = 4 Hz, 1500 pulses/session	1 session	Motor cortex	No AEs
Osou et al. (2024) [25]	NCT	N = 20 (5 females, 15 males), age = 67.6 (7.50); on standard-of-care antiparkinsonian therapy	EFD = 0.25 mJ/mm^2^, PRF = 4 Hz, 4000 pulses/session	10 sessions over 2 weeks	S1, SMA, CMA	35% no AEs, 65% side effects (fatigue, headache, dizziness)
Major depressive disorder						
Cheung et al. (2023a) [26]	RCT	N = 30 (22 females, 8 males), age = 36.55 (15.77); 23 on antidepressants, 7 unmedicated	EFD = 0.20–0.25 mJ/mm^2^, PRF = 3–4 Hz, 300 pulses/session	6 sessions over 2 weeks	dlPFC	NR
Autism spectrum disorder						
Cheung et al. (2023b) [27]	RCT	N = 32 (5 females, 27 males), age = 13.16 (1.96); on stable psychotropic medication ≥ 3 months	EFD = 0.20–0.25 mJ/mm^2^, PRF = 3–4 Hz, 800 pulses/session	6 sessions over 2 weeks	rTPJ	84% no AEs, 16% headache
Attention-deficit hyperactivity disorder						
Cheung et al. (2024) [28]	RCT	N = 32 (7 females, 25 males), age = 13.04 (1.43); all on ADHD medication	EFD = 0.20–0.25 mJ/mm^2^, PRF = 4 Hz, 800 pulses/session	6 sessions over 2 weeks	Left dlPFC	91% no AEs, 9% headache

Studies marked with * are based on the same dataset. Abbreviations: A = adverse event, CMA = cingulate motor area, DMN = default mode network, dlPFC = dorsolateral prefrontal cortex, EDF = energy flux density, IFC = inferior frontal cortex, LPC = lateral parietal cortex, NCT = non-controlled trial, NR = not reported, NRT = non-randomized trial, OFC = orbitofrontal cortex, PRF = pulse repetition frequency, RCT = randomized controlled trial, rTPJ = right temporoparietal junction, S1 = primary somatosensory cortex, SMA = supplementary motor area.

**Table 3:** Overview of the currently registered clinical trials using TPS in psychiatric and neurological disorders.

Clinical Trial Number	Patient Diagnosis	Primary Outcome Measure(s)
NCT06730438	Alzheimer’s disease	Change in ADAS Cog
NCT06681610	Amyotrophic lateral sclerosis (ALS)	Change in short intracortical inhibition of the motor cortex Change in the ALS functional rating scale
NCT05551585	Major depressive disorder	Change in Montgomery-Åsberg Depression Rating Scale
NCT06676995	Parkinson’s disease	Change in UPDRS
NCT05910619	Mild dementia	Change in ADAS Cog
NCT05602467	Mild cognitive impairment	Change in MoCA
NCT06313944	Alzheimer’s disease	Number of (serious) adverse events Number of adverse device effects

Abbreviations: ADAS Cog = Cognitive subscore of the Alzheimer’s Disease Assessment Scale, ALS = amyotrophic lateral sclerosis, MoCA = Montreal Cognitive Assessment, MCI = mild cognitive impairment, UPDRS = Unified Parkinson’s Disease Rating Scale.

**Table 4:** Measurements and outcomes related to cognitive functions.

Study	Measure	n	Pre (M ± SD)	Post (M ± SD)	FU1 (M ± SD)	FU3 (M ± SD)
Beisteiner et al. (2020) [19]	CERAD CTS	35	65.60 ± 17.66	72.52 ± 18.91	72.58 ± 21.13	72.17 ± 21.48
	LR	31	−3.36 ± 4.83	−1.45 ± 4.98	−0.29 ± 5.20	0.05 ± 5.48
	MEMORY	30	−0.31 ± 1.03	0.01 ± 1.02	0.16 ± 1.03	0.31 ± 0.83
	VERBAL	30	−0.21 ± 0.84	0.12 ± 0.92	0.16 ± 0.92	−0.21 ± 0.86
	FIGURAL	30	0.11 ± 1.07	0.03 ± 0.97	0.08 ± 1.17	−0.21 ± 0.86
Cont et al. (2022) [23]	ADAS Cog	11	25.80 ± 10.77	23.30 ± 10.27	NA	NA
	MMSE	11	17.64 ± 7.74	18.00 ± 7.12	NA	NA
	MoCA	11	11.73 ± 6.20	12 ± 6.68	NA	NA
Matt et al. (2025) [38]	CERAD CTS	60	70.93 ± 14.27	72.69 ± 13.03	72.92 ± 14.50	73.15 ± 14.90
	ADAS-Cog	60	16.77 ± 6.38	17.18 ± 6.97	16.70 ± 6.22	16.25 ± 6.03
	Clock drawing test	60	4.72 ± 2.14	4.63 ± 2.20	4.85 ± 2.17	4.75 ± 2.05
Radjenovic et al. (2025) [11]	CERAD CTS	58	56.56 ± 18.56	58.65 ± 19.44	NA	NA
Shinzato et al. (2024) [40]	ADAS-Cog	10	24.83 ± 10.26	NA	22.50 ± 9.31	21.33 ± 6.97

Abbreviations: ADAS cog = Cognitive subscale of Alzheimer’s Disease Assessment Scale, CERAD = Consortium to Establish a Registry for Alzheimer’s Disease, FU1 = 1-month follow-up, FU3 = 3-month follow-up, LR = Logistic Regression, MMSE = Mini-Mental State Examination, MoCA = Montreal Cognitive Assessment, NA = missing value.

**Table 5:** Measurements and outcomes related to mental health effects of TPS interventions in patients with AD.

Study	Measure	n	Pre (M ± SD)	Post (M ± SD)	FU1 (M ± SD)	FU3 (M ± SD)
Beisteiner et al. (2020) [19]	BDI	25	6.04 (5.40)	3.64 (3.60)	2.92 (3.64)	3.08 (3.66)
Cont et al. (2022) [23]	ADAS–depressive symptoms subscale	11	0.70 (1.10)	0.20 (0.40)	NA	NA
Matt et al. (2025) [38]	BDI	58	6.79 (5.25)	6.1 (5.27)	4.72 (4.06)	5.31 (4.96)

## Data Availability

No new data were created or analyzed in this study.

## Supplementary Materials

The following supporting information can be downloaded at: https://www.mdpi.com/article/10.3390/neurolint17110188/s1, Table S1: PRISMA checklist.

## Appendix A. Used Search Strings

1. MEDLINE: ((“transcranial pulse stimulation” OR “TPS” OR “transcranial ultrasound pulse stimulation” OR “ultrasound neuromodulation” OR “transcranial stimulation”) AND (“Alzheimer’s disease” OR “Parkinson’s disease” OR “depression” OR “autism” OR “ADHD” OR “neurocognitive disorders” OR “cognitive impairment” OR “neurodegenerative diseases” OR “mild cognitive impairment” OR “attention deficit hyperactivity disorder” OR “neuropsychiatric disorders”) AND (“clinical trial” OR “randomized controlled trial” OR “RCT” OR “pilot study” OR “observational study” OR “neurology” OR “neurophysiology” OR “neuropsychology”)) NOT (systematic review OR meta-analysis)
2. Google Scholar: (“transcranial pulse stimulation” OR “transcranial ultrasound pulse stimulation” OR „ultrasound neuromodulation”) AND (“Alzheimer’s disease” OR “Parkinson’s disease” OR “depression” OR “autism” OR “ADHD” OR “neurodegenerative diseases”) NOT (systematic review OR meta-analysis)
3. CENTRAL: *(“transcranial pulse stimulation” OR “TPS” OR “transcranial ultrasound pulse stimulation” OR “ultrasound neuromodulation” OR “transcranial stimulation”) AND (“Alzheimer* disease” OR “Parkinson* disease” OR “depress*” OR “autism” OR “ADHD” OR “neurocognitive disorders” OR “cognitive impairment*” OR “neurodegenerat* diseases” OR “mild cognitive impairment*” OR “attention deficit hyperactivity disorder” OR “neuropsychiatric disorders”) AND (“randomized controlled trial” OR “RCT” OR “clinical trial” OR “pilot study”) NOT (“systematic review” OR “meta-analysis”)
4. PsycInfo: ((“transcranial pulse stimulation” OR “TPS” OR “transcranial ultrasound pulse stimulation” OR “ultrasound neuromodulation” OR “transcranial stimulation”) AND (“Alzheimer’s disease” OR “Parkinson’s disease” OR “depression” OR “autism” OR “ADHD” OR “neurocognitive disorders” OR “cognitive impairment” OR “neurodegenerative diseases” OR “mild cognitive impairment” OR “attention deficit hyperactivity disorder” OR “neuropsychiatric disorders”) AND (“clinical trial” OR “randomized controlled trial” OR “RCT” OR “pilot study” OR “observational study” OR “neurology” OR “neurophysiology” OR “neuropsychology”)) NOT (systematic review OR meta-analysis)
5. Web of Science: ((“transcranial pulse stimulation” OR “TPS” OR “transcranial ultrasound pulse stimulation” OR “ultrasound neuromodulation” OR “transcranial stimulation”) AND (“Alzheimer* disease” OR “Parkinson* disease” OR “depress*” OR “autism” OR “ADHD” OR “neurocognitive disorders” OR “cognitive impairment*” OR “neurodegenerat* diseases” OR “mild cognitive impairment*” OR “attention deficit hyperactivity disorder” OR “neuropsychiatric disorders”) AND (“clinical trial” OR “randomized controlled trial” OR “RCT” OR “pilot study” OR “observational study” OR “neurology*” OR “neurophysiology*” OR “neuropsychology*”)) NOT (“systematic review” OR “meta-analysis”)
